# The Protective Effect of Luteolin in Glucocorticoid-Induced Osteonecrosis of the Femoral Head

**DOI:** 10.3389/fphar.2020.01195

**Published:** 2020-08-12

**Authors:** Zijian Yan, Jingdi Zhan, Weihui Qi, Jian Lin, Yijiang Huang, Xinghe Xue, Xiaoyun Pan

**Affiliations:** ^1^Department of Orthopaedics, The Second Affiliated Hospital and Yuying Children’s Hospital of Wenzhou Medical University, Wenzhou, China; ^2^Department of Orthopaedics, Zhejiang Provincial Key Laboratory of Orthpaedics, Wenzhou, China; ^3^The Second School of Medicine, WenZhou Medical University, Wenzhou, China

**Keywords:** glucocorticoid-induced osteonecrosis of the femoral head, luteolin, STAT1/caspase3 pathway, apoptosis, mitochondrial pathway

## Abstract

Glucocorticoid-induced osteonecrosis of the femoral head (GIONFH) is a frequently occurring type of nontraumatic osteonecrosis. A failure of the timely treatment can eventually result in the collapse of the subchondral bone structure. Luteolin (Lut), a compound extracted from Rhizoma Drynariae, is reported to possess multiple pharmacological properties including anticancer, antioxidant, antiapoptosis, and antiinflammatory properties. However, whether Lut has a protective effect on the development of GIONFH remains unclear. In this study, we evaluated the effect of Lut on Dexamethasone (Dex)-induced STAT1/caspase3 pathway *in vitro* and evaluated GIONFH model *in vivo*. *In vitro*, Lut inhibited the upregulation of Dex-induced phospho-STAT1, cleaved caspase9, and cleaved caspase3. In addition, Lut inhibited Dex-induced expression of Bax and cytochrome c and increased the expression of B cell lymphoma-2(Bcl-2). *In vivo*, Lut decreased the proportion of empty lacunae in rats with GIONFH. Taken together, these findings indicate that Lut may have therapeutic potential in the treatment of GIONFH. Further, this effect might be achieved by suppressing mitochondrial apoptosis of osteoblasts *via* inhibition of STAT1 activity.

## Introduction

Osteonecrosis of the femoral head (ONFH) is a progressive orthopedic disease, including traumatic and nontraumatic osteonecrosis (Nazal et al., 2019). Glucocorticoid-induced ONFH (GIONFH) is a frequently occurring type of nontraumatic osteonecrosis, which can eventually result in the collapse of the subchondral bone structure if not treated timely (Kerachian et al., 2009; Deng et al., 2019). However, specific mechanisms causing GIONFH remain uncertain. It is increasingly evident that the effects of glucocorticoids on normal bone metabolism may be the main cause of GIONFH (Kuang et al., 2019). Increase in the apoptosis of osteoblasts and osteocytes, prolongation of the life span of osteoclasts and endothelial cell apoptosis, and inhibition of osteoblast and osteoclast precursor production are all directly affected by the use of glucocorticoids (Kerachian et al., 2009; Weinstein et al., 2017). Osteoblasts, as the main target of glucocorticoids, play important roles in the process of bone formation; further, dexamethasone (Dex) has been reported to induce apoptosis of mouse osteoblasts (Brennan-Speranza et al., 2012; Gamez et al., 2016; Gu et al., 2019).

Caspase3 is an aspartic acid-specific cysteine protease, the activation of which could participate in apoptotic cell death (Cheng et al., 2014). Furthermore, B-cell lymphoma-2 (Bcl-2), cytochrome c, Bcl-2-associated X (Bax), cleaved caspase3 (C- caspase3), and cleaved caspase9 (C-caspase9) proteins have also been proved to regulate apoptosis (Liu et al., 2012). Signal transducer and activator of transcription 1 (STAT1) is a member of the STAT protein family, which has a pro-apoptotic effect (Butturini et al., 2018). Furthermore, a previous study has shown that the STAT1/caspase3 pathway is closely associated with GIONFH (Xu et al., 2014).

Rhizoma Drynariae, a traditional Chinese herbal medicine, is used for treating bone diseases such as osteoporosis and osteoporotic fractures (Kang et al., 2014; Gan et al., 2019). Further, luteolin (Lut), a compound extracted from Rhizoma Drynariae, is reported to possess multiple pharmacological properties including anticancer, antioxidant, antidiabetic, and antiinflammatory properties (Zang et al., 2016; Al-Megrin et al., 2019; Gao et al., 2019; Li et al., 2019). Previous studies have shown that Lut exerts its antiapoptotic effects the through mitochondrial signaling pathway and downregulates caspase3 and caspase9 expression (Yu et al., 2019). Furthermore, Lut significantly inhibits lipopolysaccharide (LPS)/interferon (IFN)-gamma-induced phospho-STAT1 (P-STAT1) levels (Kao et al., 2011). Additionally, Lut inhibits glucocorticoid-induced osteoporosis by regulating the GSK-3β/Lrp-5/ERK signaling pathway (Jing et al., 2019). However, it is unclear whether Lut attenuates Dex-induced osteoblast apoptosis and GIONFH development *in vivo*. Therefore, in the present study, we evaluated the effect of Lut on Dex-induced apoptosis and its potential mechanism *in vitro* and the GIONFH model *in vivo*.

## Methods

### Reagents and Antibodies

Luteolin (purity > 98%), dimethylsulfoxide (DMSO), type I collagenase, and dexamethasone were acquired from Sigma Chemical Co (St. Louis, MO, USA). Antibodies against Bax, Bcl-2, STAT1, caspase3, caspase9, C-caspase9, cytochrome c, and GADPH were obtained from Proteintech (Chicago, IL, USA). Goat anti-rabbit IgG-HRP was obtained from Bioworld (Dublin, OH, USA) and primary antibodies against P-STAT1, and C-caspase3 were acquired from Cell Signaling Technology (MA, USA). Cell-Counting Kit-8 (CCK-8) was purchased from Dojindo (Kumamo, Japan). The Cell Apoptosis-DNA enzyme linked immunosorbent assay (ELISA) Plus kit was obtained from Roche (Palo Alto, CA, USA). Bovine serum albumin (BSA), fetal bovine serum (FBS), and alpha-minimal essential medium (α-MEM) were purchased from Healthcare life Sciences (Hyclone; Logan, UT, USA).

### Cell Culture

Calvariae were obtained from newborn rats (within 1 day of birth; Animal Center of the Chinese Academy of Sciences, Shanghai, China) following euthanization using pentobarbital. All skull tissues were harvested from the rats under aseptic conditions, separated from other the connective tissue, chopped into 1 × 1 mm^2^ pieces, rinsed with phosphate buffer saline (PBS) three times, and thereafter digested in 0.25% trypsin–EDTA solution for 30 min. Following this, the tissues were incubated with 2 mg/ml (0.1%) collagenase I for 1 h in an incubator maintained at 5% CO2 at 37°C. The tissue digestate was centrifuged at 1000 rpm for 5 min, after which the supernatant was discarded and the osteoblasts were suspended in α-MEM supplemented with 1% penicillin/streptomycin and 10% FBS. The osteoblasts were passaged when they were 80% to 90% confluent, after dissociation with a 0.25% trypsin-EDTA solution. Only passage 0 to 2 cells were used to ensure no loss of phenotype in this study.

### Cell Viability

The effect of Lut on cell viability was evaluated *via* the CCK-8 assay, in accordance with the manufacturer’s instructions. The optical density of the solution in each well was measured at 450 nm using a spectrophotometer from ThermoFisher(Waltham, MA,USA).

### ELISA

The Cell Apoptosis-DNA ELISA Plus kit (Palo Alto, CA, USA) was used to analyze the degree of apoptosis of the osteoblasts.

### LDH Release Assay

LDH release assay was used to assess Dex-induced cytotoxicity. Osteoblasts were treated with Dex (1 μM) and subsequently incubated with different concentrations of Lut (0–10 μM) for 24 h in an incubator maintained at 37°C with 5% CO2. The cell culture medium was collected and LDH activity was measured in accordance with the manufacturer’s instructions (Beyotime, Nanjing, China).

### Western Blotting

The protein levels of STAT1, P-STAT1, caspase3, caspase9, C-caspase3, C-caspase9, cytochrome c, Bax, GADPH, and Bcl-2 were detected *via* Western blotting. Total cellular proteins were isolated using a radio immunoprecipitation assay (RIPA) lysis buffer [with 1% phenylmethane sulfonyl fluoride (PMSF)] and the protein concentration was subsequently quantified using the bicinchoninic acid assay (BCA) protein assay kit (Beyotime). Herein, 30-ng aliquots of total protein were separated *via* 10%–15% sodium dodecyl sulfate-polyacrylamide gel electrophoresis (SDS-PAGE) and then transferred onto polyvinylidene difluoride membranes (Bio-Rad, CA, USA). After blocking with 5% nonfat dried milk for 2 h at room temperature (RT), the membranes were promptly rinsed three times with tris-buffered saline with Tween (TBST). Thereafter, the membranes were probed overnight with primary antibodies (1:1,000) at 4°C, followed by rinsing with TBST three times (5 min each time) and incubation with an appropriate secondary antibody (1:3,000) for 2 h at RT. The bands were visualized using electrochemiluminescence plus reagent (Invitrogen, Carlsbad, CA, USA) and the intensity of the blots was then quantified using the Image Lab 3.0 software (Bio-Rad, Hercules, CA, USA).

### Immunofluorescence Analysis

Osteoblasts were cultured on glass coverslips in a six-well plate and then incubated overnight in a conditioned medium. The cells treated with or without Lut (10 μM) for 2 h and then coincubated in the absence or presence of Dex (1 μM) for 24 h. Subsequently, the osteoblasts were washed three times with PBS and then permeabilized with the 0.5% Triton X-100 (Solarbio Science & Technology, Beijing, China) for 10 min, following which they were fixed with 4% paraformaldehyde for 15 min. The cells were then blocked with 5% goat serum for 1 h at 37°C, rinsed three times with PBS, stained with antibodies against P-STAT1 (1:200) and C- caspase3 (1:200) at 4°C overnight, and then stained with Alexa Fluor^®^594 or Fluor^®^488-conjugated secondary antibody (1:400) for 1 h. Following this, the cells were stained by DAPI (Invitrogen) for 1 min. Finally, the cells were imaged *via* fluorescence microscopy (Olympus Inc, Tokyo, Japan). In addition, the fluorescence intensity was evaluated by researchers blinded to the experimental groups using Image J software 2.1 (NIH, Bethesda, MD, USA).

### TUNEL Assay

Apoptotic osteoblasts were detected using the TUNEL Assay Kit, in accordance with the manufacturer’s instructions. The cultured osteoblasts were collected on glass coverslips in a 6-well plate. The osteoblasts were fixed with 4% paraformaldehyde for 20 min, rinsed with PBS three times, incubated with 3% H_2_O_2_, permeabilized with 0.1% Triton X-100 for 10 min, and thereafter stained using *in situ* cell death detection kit (Hoffmann-La Roche Ltd., Basel, Switzerland), followed by DAPI staining for 1 min. The apoptotic cells were imaged *via* fluorescence microscopy (Olympus).

### Animal Model of GIONFH

Twelve-week-old Sprague–Dawley rats were acquired from the Animal Center of the Chinese Academy of Sciences, Shanghai, China, authorized by the Animal Care and Use Committee of Wenzhou Medical University. In all, 30 rats were divided into three groups on average: control, model, and Lut groups. The model and Lut group rats was administered intramuscular injections of 10 mg/kg Dex twice a week for 8 weeks. Meanwhile, the Lut group rats were followed by an intragastric administration of 10 mg/kg Lut twice a week for 8 weeks. The control group was injected with normal saline (NS). The femoral heads of rats were collected after 8 weeks, and their histology was studies by hematoxylin-eosin staining (HE staining), immunohistochemical analysis, and image analysis of micro-computed tomography (CT) images.

### Micro-Computed Tomography

The specimens were scanned on a miniature CT system (70 kV, 114 μA; micro-CT 80 scanner; Scanco Medical, Bassersdorf, Switzerland). The diagnostic criteria for ONFH were as follows: flattened shape of the femoral head, cyst degeneration, fracture of trabecula, and sclerosis band (Dong Y. et al., 2015). Proximal femur was quantitatively calculated as the region of interest. The trabecular number (Tb.N), trabecular separation (Tb.Sp), trabecular thickness (Tb.Ts), and bone volume/total volume (BV/TV) were calculated using the InveonTM analysis workstation (Munich, Germany) (Low et al., 2014).

### Histological Analysis

The femoral head samples were fixed in 4% paraformaldehyde (Sigma Chemical Co) at 4°C for 24 h. Following this, the samples were transferred 10% EDTA solution (Solarbio Science & Technology) for decalcification for 4 weeks. Finally, the samples were dehydrated and embedded in paraffin blocks. The blocks were then sliced into 5-μm sections. Femoral head samples underwent HE staining and were observed under a microscope (Leica Microsystems, Wetzlar, Hesse, Germany) to identify empty lacunae, pyknotic nuclei of osteocytes, and broken bone trabeculae. The proportion of empty lacunae was assessed by established criteria as described earlier (Yamamoto et al., 1997).

### Immunohistochemical Analysis

Paraffin-embedded 5-μm tissue sections were dewaxed with xylene, hydrated with an ethanol concentration gradient, treated with 3% hydrogen peroxide, and then incubated with 0.4% pepsin at 37°C for 30 min. The slices were blocked with 10% goat serum at RT for 30 min; following this, they were incubated overnight with C-caspase3 primary antibody (1:200) at 4°C and then with HRP-conjugated secondary antibody and diaminobenzene (DAB) (Solarbio Science & Technology). The slices were stored at 4°C.

### Statistical Analysis

The data are expressed as means ± standard deviation (SD) of at least three independent trials. Statistical Product and Service Solutions (SPSS) (Chicago, IL, USA) were performed for all statistical analyses. The data were analyzed followed by Tukey’s test to compare control and treatment groups. For statistical comparison of more than two groups, data were evaluated using one-way ANOVA. P values < 0.05 were considered significant.

## Results

### Potential Cytotoxicity of Lut on Osteoblasts

**Figure 1:**
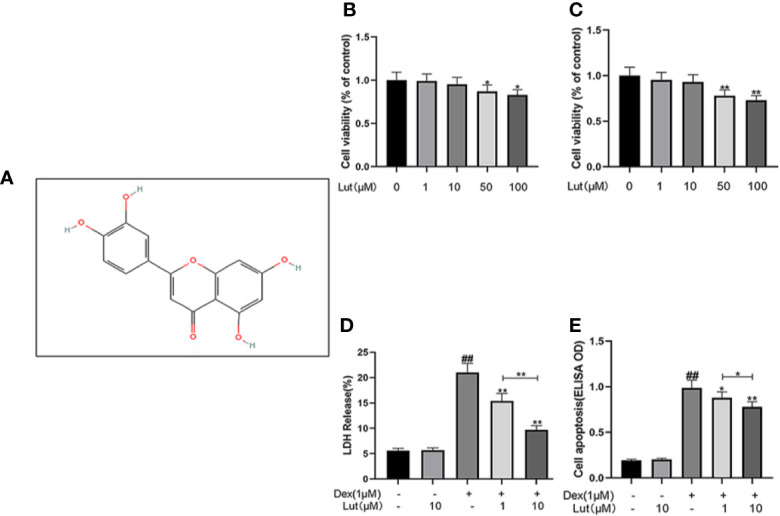
Potential Cytotoxicity of Lut on osteoblasts. Chemical structure of Lut **(A)**. The cytotoxic effects of Lut on osteoblasts were determined with increasing concentrations (0, 1, 10, 50 and 100 μM) for 24 and 48 hours using a CCK8 assay **(B**, **C)**. And the viability of osteoblasts induced by Dex was determined by ELISAs and LDH release assay **(D**, **E)**. Values represent the averages ± S.D. Significant differences between different groups are indicated as ^##^P < 0.01, vs control group; *P < 0.05, **P < 0.01, vs Dex alone treatment group, n = 5.

The chemical structure of Lut is shown in **Figure 1A**. Osteoblasts were cultured with Lut at different concentrations (0, 1, 10, 50, and 100 μM). As showed in **Figures 1B, C**, Lut was not cytotoxic to osteoblasts at concentrations of 1 and 10 μM at 24 and 48 h. And cotreatment of osteoblasts with different concentrations of Lut could significantly ameliorate Dex-induced cell death in a concentration-dependent manner (**Figure 1D**). Furthermore, ELISA further confirmed that Lut had a protective effect on apoptosis induced by Dex (**Figure 1E**).

### Effects of Lut on Dex-Induced STAT1/caspase3 Pathway in Rat Osteoblasts

**Figure 2:**
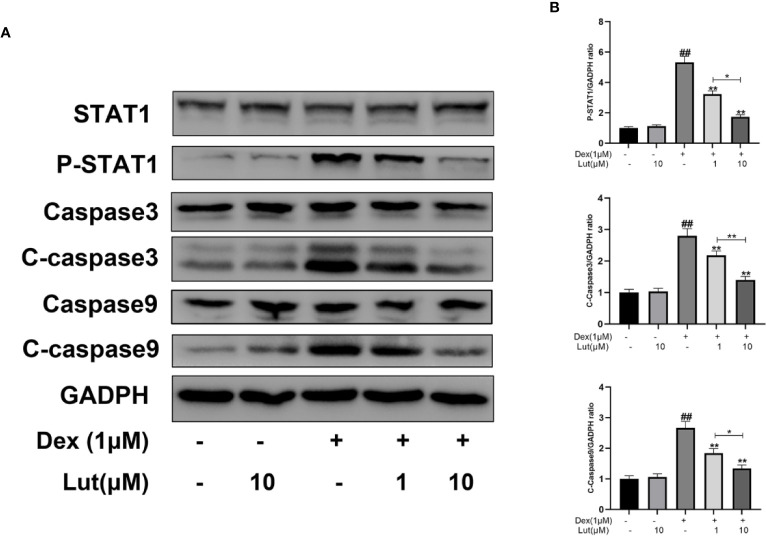
Effects of Lut on Dex-induced STAT1/caspase3 pathway in Rat Osteoblasts. The osteoblasts treated with or without Lut (1、10 μM) for 2 h and then coincubated in the absence or presence of Dex (1 μM) for 24 h. The protein expression of STAT1, P-STAT1, caspase3, cleaved-caspase3, caspase9 and cleaved-caspase9 were measured by western blot **(A)** and quantification of the resulting bands **(B)**. Significant differences between different groups are indicated as ^##^P < 0.01, vs. control group; *P < 0.05, **P < 0.01 vs. Dex alone treatment group, n = 5.

To further evaluate the effects of Lut on STAT1/caspase3 pathway in osteoblasts, the expression levels of STAT1, P-STAT1, caspase3, C-caspase3, caspase9, and C-caspase9 were measured by Western blotting. Following stimulation with Dex for 24 h, P-STAT1, C-caspase3, and C-caspase9 levels increased considerably. However, pretreatment with Lut for 2 h, inhibited the upregulation of Dex-induced P-STAT1, C-caspase3, and C-caspase9 (**Figure 2**).

### Lut Inhibited the DEX-Induced Expression of P-STAT1 in the Nucleus and C-Caspase3 in the Cytoplasm

**Figure 3:**
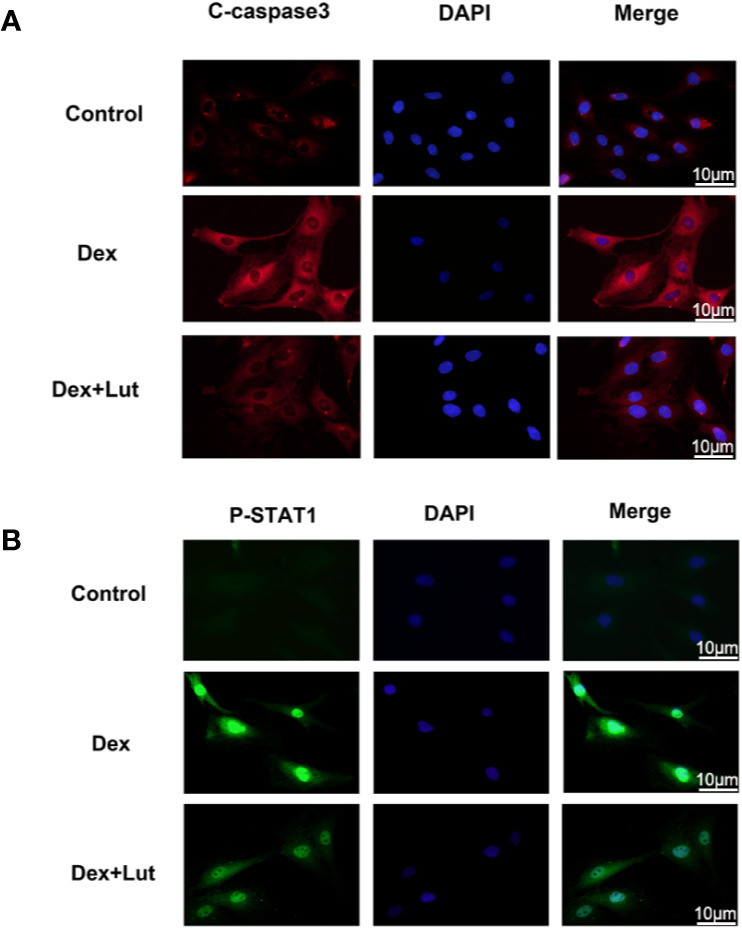
Up-regulation STAT1 and cleaved caspase-3 expression in osteoblasts induced by Dex. The osteoblasts treated with or without Lut (10 μM) for 2 h and then coincubated in the absence or presence of Dex (1 μM) for 24 h. Typical P-STAT1 **(A)** and cleaved caspase‐3 **(B)** were detected by immunofluorescence combined with DAPI staining for nuclei (scale bar: 10 μm). n = 5.

To further study on the effect of STAT1 on the expression of caspase-3, the osteoblasts were cultured with different concentrations in Lut (10 μM for 2h) before stimulation with Dex (1 μM for 24 h).Immunofluorescence analysis showed that in the control group, there was almost no expression of P-STAT1 in the nucleus and C-caspase3 in the cytoplasm, but this significantly expressed induced by Dex. Further, treatment with Lut inhibited Dex-induced C-caspase3 and P-STAT1 expression (**Figures 3A, B**), which further confirmed Lut inhibited the Dex-induced activation of apoptosis *via* the STAT1/caspase3 signaling pathway.

### Effects of Lut on Dex-Induced Mitochondrial Apoptosis Pathway in Rat Osteoblasts

**Figure 4:**
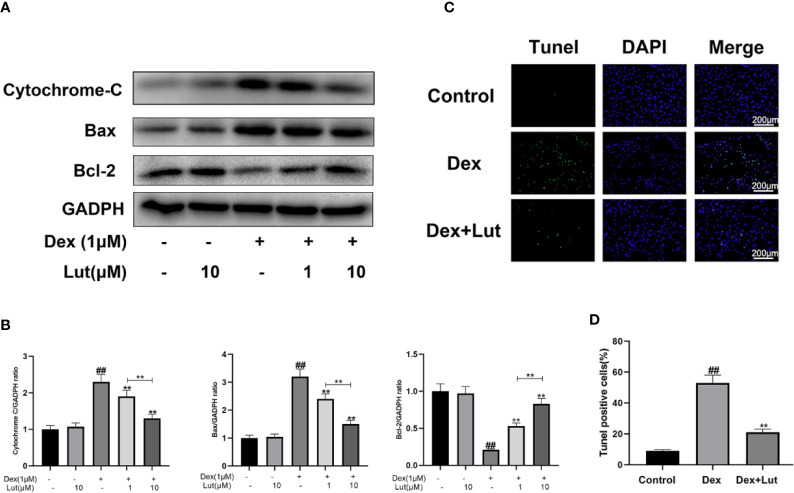
Effect of Lut on mitochondrial apoptosis pathway of rat osteoblasts induced by Dex. The protein expressions of Bcl-2, Bax and cytochrome c in osteoblasts treated above were detected by Western blotting **(A)** and quantification of the resulting bands **(B)**. Apoptotic osteoblasts were detected by utilizing with a Tunel Assay Kit. TUNEL assay was performed in osteoblast cells. The green fluorescence represents the apoptosis of osteoblasts and blue fluorescence represents the nucleus **(C)** (scale bar: 200 μm). Image J software 2.1 to evaluate the number of TUNEL positive cells **(D)**. Significant differences between different groups are indicated as ^#^P < 0.01, vs. control group; *P < 0.05, **P < 0.01, vs. Dex alone treatment group, n = 5.

To explore whether Dex-induced mitochondrial apoptosis in osteoblasts was inhibited by Lut, the osteoblasts were cultured with different concentrations in Lut (1 and 10 μM for 2h) before stimulation with Dex (1 μM for 24 h). We performed Western blotting to detect the protein expression levels of Bax, cytochrome c, and Bcl-2. As shown in **Figures 4A, B**, the expression level of cytochrome c and Bax significantly increased, whereas the protein expression levels of Bcl-2 decreased in the Dex group. However, pretreatment with Lut led to enhanced Bcl-2 expression and reduced cytochrome c and Bax expression after treatment with Dex in a dose-dependent manner. Further, no difference was observed in the expression levels of cytochrome c, Bax, and Bcl-2 between the Lut and control groups.

### TUNEL Assay

Consistent with the trends observed *via* ELISA, the results of TUNEL assay showed that the apoptosis rate was significantly increased in the Dex group, which confirmed that the Dex-induced death of osteoblasts occurred in the form of apoptosis. And Lut pretreatment significantly reduced the apoptosis induced by Dex (**Figures 4C, D**), which showed that Lut plays an antiapoptosis role in osteoblasts.

### Sample Handling

**Figure 5:**
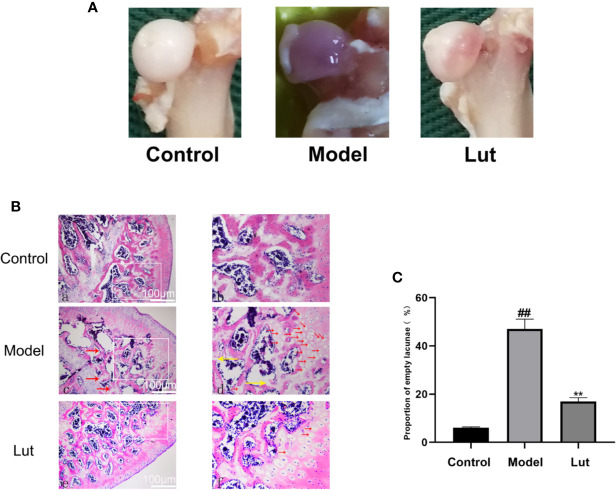
The surface and histological analysis of glucocorticoid-induced osteonecrosis of the femoral head *in vivo*. In the control group, the femoral head had a white appearance. However, the femoral head surface was cyanotic in the model group. In the Lut group, the appearance of femoral head was between that of the control and model groups **(A)**. And there were no empty lacunae in the femoral head of the control group. A large number of empty bone lacunae and necrotic bone marrow cells were seen in the model group, while there were few empty bone lacunae in the Lut group **(B)** (scale bar: 100 μm). The proportion of empty bone lacunae in the model group was significantly higher than that in the control group and Lut group **(C)**. Values represent the averages ± S.D. Significant differences between different groups are indicated as ^##^P < 0.01 vs. the Control group and ^**^P < 0.01 vs. the Model group.

As shown in **Figure 5A**, the femoral head had a white alabaster appearance and an even surface in the control group. Conversely, an obvious cyanotic surface of the femoral head was observed in the model group, which suggested that there is hemorrhage and necrosis of the femoral head (Dong Y. L. et al., 2015; Lin et al., 2019). In the Lut group, the femoral head showed an intermediate appearance compared with the two groups mentioned above.

### Lut Mitigates the Progression of GIONFH in Rat Model

In GIONFH, bone trabeculae are permeated by the pyknotic nuclei of osteocytes or empty lacunae with surrounding bone marrow cells (Yamamoto et al., 1997; Steffen et al., 2010). In our study, 2 of 10 rats in the Lut group and 8 of 10 rats in the model group developed osteonecrosis, the incidence of GIONFH was significantly decreased in the Lut group compared with that in the model group. As shown in **Figures 5B, C**, in the control group, none of the rats developed osteonecrosis. The model group showed a significantly increased proportion of empty lacunae on HE staining compared with the control group. Conversely, lower proportions of empty lacunae were observed in the Lut group.

### Results of Micro-CT

**Figure 6:**
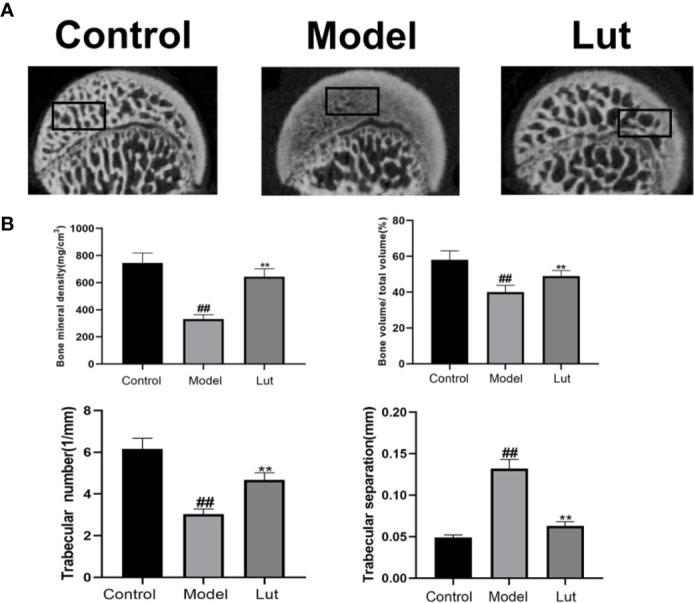
Analysis of bone structure of rat femoral head by Micro-CT scanning. The Micro-CT scanning of the femoral head and quantitative analysis of bone trabeculae was compared among the control group, the model group and the Lut group **(A, B)**. Values represent the averages ± S.D. Significant differences between different groups are indicated as ^##^P < 0.01 vs. the Control group and ^**^P < 0.01 vs. the Model group.

Micro-CT was used to analyze bone tissues in the femoral heads. In the model group, GIONFH changes were obvious and the subchondral trabeculae were seriously damaged (**Figure 6A**). The microstructural parameters, such as BMD, Tb.N, and BV/TV, were increased significantly and Tb.Sp was significantly reduced in the Lut group compared with those in the model group (**Figure 6B**). In conclusion, Lut could significantly reverse Dex-induced bone loss.

### Effect of Lut on C-Caspase3 *In Vivo*

**Figure 7:**
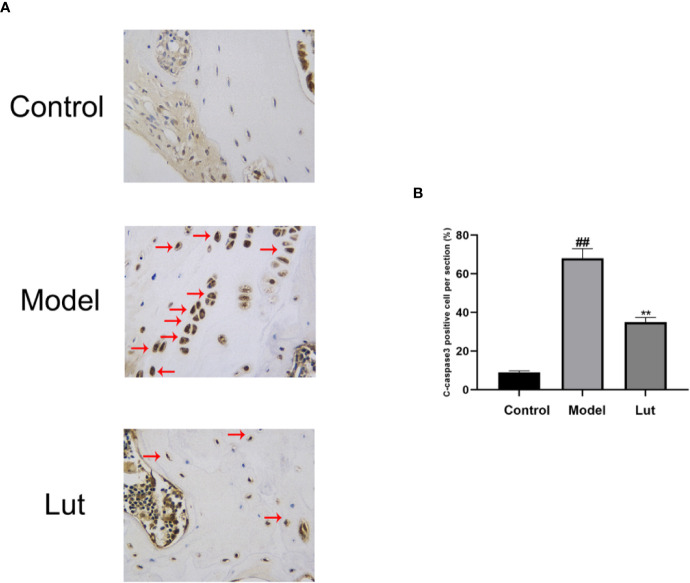
The effect of Lut on the activation of C-caspase3 *in vivo* Immunohistochemical staining of C-caspase3 expression in the samples. Caspase3 is an aspartic acid-specific cysteine protease, the activation of which could participate in apoptotic cell death. Positive expressions was found in the model groups (scale bar: 10 μm) **(A)**. The percentages of Cleaved-caspase3 positive cells in each section were quantified by Image J software **(B)**. Values represent the averages ± S.D. Significant differences between different groups are indicated as ^##^P < 0.01 vs. the Control group and **P < 0.01 vs. The Model group.

To investigate the effect of Lut on C-caspase3 *in vivo*, the expression levels of C-caspase3 proteins were detected by immunohistochemical analysis. Only a small amount of positive expression was found in the control group. In contrast, a higher proportion of C-caspase3-positive cells were observed in the model group, and Lut could attenuate the abovementioned effect induced by Dex (**Figure 7**).

## Discussion

With the widespread clinical application of Dex and other glucocorticoids, GIONFH has become a frequently occurring type of nontraumatic femoral head necrosis (Xu et al., 2018). Although GIONFH pathogenesis still remains uncertain, several factors such as cell differentiation disorder, angiogenesis hindrance, apoptosis, adipogenesis and fatty deposition have been postulated to play a role in GIONFH (Han et al., 2010a; Han et al., 2010b; Deng et al., 2018). Apoptosis of osteoblasts is an important factor leading to the induction of ONFH by glucocorticoids *via* the activation of the glucocorticoid receptor (Yun et al., 2009; Nie et al., 2019). Further, previous studies have shown that Dex can induce osteoblast apoptosis and GIONFH *via* STAT1/caspase3 pathways (Feng et al., 2017). Thus, it may be possible to prevent GIONFH by the inhibition of osteoblast apoptosis. Lut, a compound extracted from Rhizoma Drynariae, has been found to have antioxidant, anticancer, antidiabetic, antiapoptosis, and antiinflammatory effects. In this study, we evaluated the effect of Lut on Dex-induced apoptosis of osteoblasts and the potential mechanism involved *in vitro* and *in vivo*.

In this study, we first used CCK-8 to analyze the potential toxic concentration of the Lut on osteoblasts. And LDH release assay showed that Lut could significantly ameliorate Dex-induced cell death in a concentration-dependent manner. Then Cell Apoptosis-DNA ELISA and TUNEL staining confirmed that the Dex-induced death of osteoblasts occurred in the form of apoptosis. Furthermore, we found that the expression levels of cytochrome c and Bax significantly increased, whereas the protein production of Bcl-2 was reduced in the Dex group, which showed that Lut plays an antiapoptosis role through the mitochondrial pathway in osteoblasts.

Two classical apoptotic pathways have been identified, including intrinsic mitochondria and extrinsic death receptor (Rao et al., 2004; Zeng et al., 2012). The mitochondrial pathway is characterized by mitochondrial membrane depolarization, which results in the release of cytochrome c, followed by caspase3 activation (Yang et al., 2010). As a family of specific cysteine proteases and caspases can cleave substrates located in intracellular compartments that are initiators and executors of apoptosis, including caspase3, 6, 8, 9, and 10 (Park et al., 2007; Kopeina et al., 2018). Among these, caspase3 has been known as the death protease because of the critical role it plays in cell apoptosis, which has been reported to be activated in ONFH (Scarabelli et al., 2004; Yang et al., 2010; Abdi et al., 2011; Cui et al., 2018). The mitochondrial pathway is further characterized by mitochondrial membrane depolarization, which in turn results cytochrome c release and caspase3 activation (Yang et al., 2010). Dex, a member of the glucocorticoid family, has been considered to be an activator of the mitochondrial pathway, which plays a vital role in osteoblast apoptosis (Nie et al., 2019). After binding to Dex, the glucocorticoid receptor is activated and translocates from the cytoplasm to nucleus, transactivating related genes after binding with chromatin, which then makes the mitochondria release cytochrome c into the cytoplasm (Robertson et al., 1993). The release of cytochrome c from the mitochondrial membrane space to the cytoplasm is the key step in the mitochondria-mediated apoptosis signal transduction pathway (Ott et al., 2007). Cytochrome c induces the sequential activation of the caspase cascades, including the activation of caspase9, which then activates downstream caspase3 (Meng et al., 2012; Zhang et al., 2015). Different members of the Bcl-2 family of proteins, such as Bax, seem to control the release of cytochrome c (Wang et al., 2020; Yusuf and Casey, 2020). Bax plays a role in increasing the permeability of the mitochondrial outer membrane for releasing cytochrome c into the extracorporeal mitochondrial environment (Ott et al., 2002; Pagliari et al., 2005). Furthermore, Bax not only is the main regulator of mitochondrial outer membrane permeabilization but also is a central mediator of apoptosis (Westphal et al., 2014; Reyna et al., 2017). In contrast, Bcl-2 is a prototype antiapoptosis protein located in the mitochondria that prevents pro-apoptotic proteins such as Bax from being recruited and activated in the mitochondria (Low et al., 2011). Lut has been previously reported to exert its antiapoptotic effects through the mitochondrial signaling pathway and by downregulating caspase3 and caspase9 expressions. However, the antiapoptotic effects of Lut in GIONFH have not yet been reported. In our study, we demonstrated that Lut plays an antiapoptosis role through the mitochondrial pathway in osteoblasts.

As a key upstream protein of caspase3, STAT1 has been proved to be a pro-apoptotic factor. The inhibition of STAT1 reduces cell apoptosis and contributes to bone formation and fracture healing (Kim et al., 2003; Tajima et al., 2010). Previous studies have found that once STAT1 is activated, it can lead to the upregulation of caspase3 expression, in turn leading to the induction of apoptosis (Sironi and Ouchi, 2004; Dedoni et al., 2010). Meanwhile, low constitutive levels of caspase3 have been shown to be expressed in STAT1-null cells (Kumar et al., 1997). Furthermore, STAT1/caspase3 has been reported to play a vital role in seawater aspiration-induced acute lung injury and glucocorticoid-induced avascular necrosis of the femoral head (Liu et al., 2016). In the present study, we found that Lut inhibited the Dex-induced activation of apoptosis *via* the STAT1/caspase3 signaling pathway by Western blot and immunofluorescence analysis and Lut may have therapeutic potential in the treatment of GIONFH. Further, this effect might be achieved by suppressing mitochondrial apoptosis of osteoblasts *via* inhibition of STAT1 activity. Moreover, immunohistochemical analysis showed the effects of Lut on the activation of caspase3 *in vivo*.

To further study the protective effect of Lut *in vivo*, an animal model of GIONFH was established. Results of HE staining showed that the model group had significantly increased proportions of empty lacunae compared with the control group. And the surface of the femoral head in the model group showed obvious purplish appearance, indicating that there was hemorrhage and necrosis of the femoral head. In contrast, a lower proportion of empty lacunae was observed in the Lut group. Further, the results of micro-CT showed that GIONFH changes were obvious and the subchondral trabeculae were seriously damaged in the model group. The microstructural parameters, such as BMD, Tb.N, and BV/TV, were significantly increased and Tb.Sp was reduced in the Lut group compared with those in the model group. Taken together, Lut can ameliorate GIONFH *in vivo*.

In this study, the treatment of Lut could inhibit the Dex-induced activation of apoptosis *via* the STAT1/caspase3 signaling pathway. The clear mechanism remains to be further studied, and there are the following conjectures: (1) Lut directly weakened the binding of Dex to GR (inactivated hormone or competitively binded GR to Dex); (2) The activity of GR was reduced by Lut; (3) Lut inhibited the transfer of activated GR to the nucleus; (4) Lut or its active decomposition acted on the binding region of GR and genes, and inhibited the expression of related genes. In addition to apoptosis of osteoblasts, recent study showed excessive adipogenesis and fatty deposition has been considered to be another important mechanism of GIONFH (Han et al., 2016). And enhancing the activity and expression of P-glycoprotein can inhibit adipogenesis and promote osteogenesis. Therefore, the protective effect of Lut on adipogenesis and fatty deposition of GIONFH and effect of P-glycoprotein on osteoblasts needs to be further clarified.

## Conclusion

In conclusion, this study demonstrated that Lut inhibited the activation of apoptosis induced by Dex *via* the STAT1/caspase3 signaling pathway. Further, Lut also has a protective effect on GIONFH model rats by reducing the percentage of empty bone lacunae. The pathway for inhibiting mitochondrial apoptosis was also evaluated. Taken together, these findings indicate that Lut may have therapeutic potential in the treatment of GIONFH.

## Data Availability Statement

The raw data supporting the conclusions of this article will be made available by the authors, without undue reservation, to any qualified researcher.

## Ethics Statement

The animal study was reviewed and approved by Animal Care and Use Committee of Wenzhou Medical University.

## Author Contributions

YH, WQ: Conceptualization, Methodology. JZ: Data curation, Writing—Original draft preparation, Software. XX: Visualization, Investigation. XP: Supervision. JL: Software, Validation: ZY, XP: Writing—Reviewing and editing.

## Funding

This study was supported by Wenzhou Science and Technology Plan Project, Zhejiang Province, China (Y20180036), Science and Technology Program of traditional Chinese Medicine, Zhejiang Province, China (2019ZB076).

## Conflict of Interest

The authors declare that the research was conducted in the absence of any commercial or financial relationships that could be construed as a potential conflict of interest.
