# Supplementary figures and images for: The Protective Effect of Luteolin in Glucocorticoid-Induced Osteonecrosis of the Femoral Head
Source: Front Pharmacol. 2020 Aug 12;11:1195. doi: 10.3389/fphar.2020.01195 (PMC7435053; doi:10.3389/fphar.2020.01195)

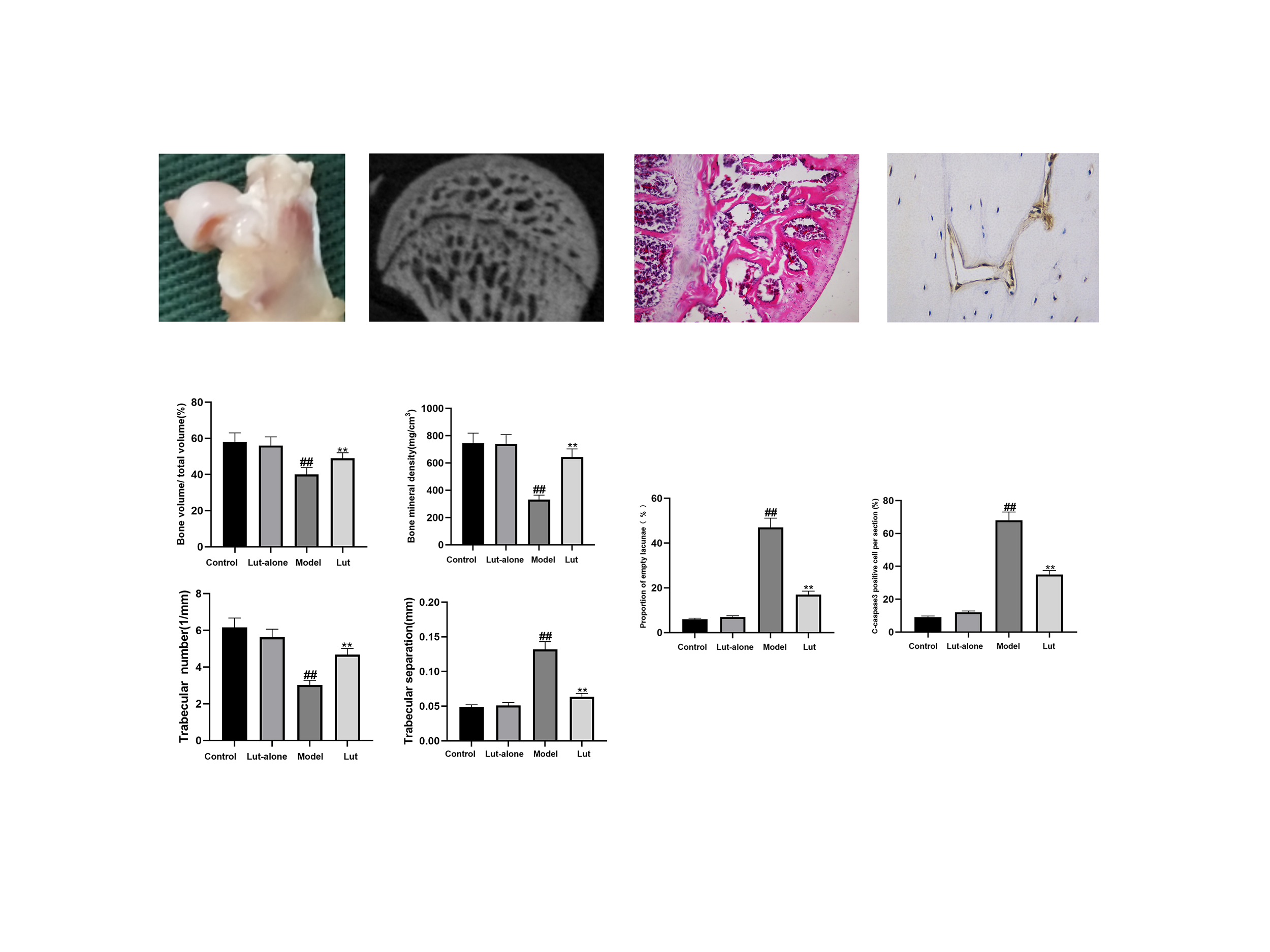

Supplement: Supplementary file 1 [file Image_1.tif]

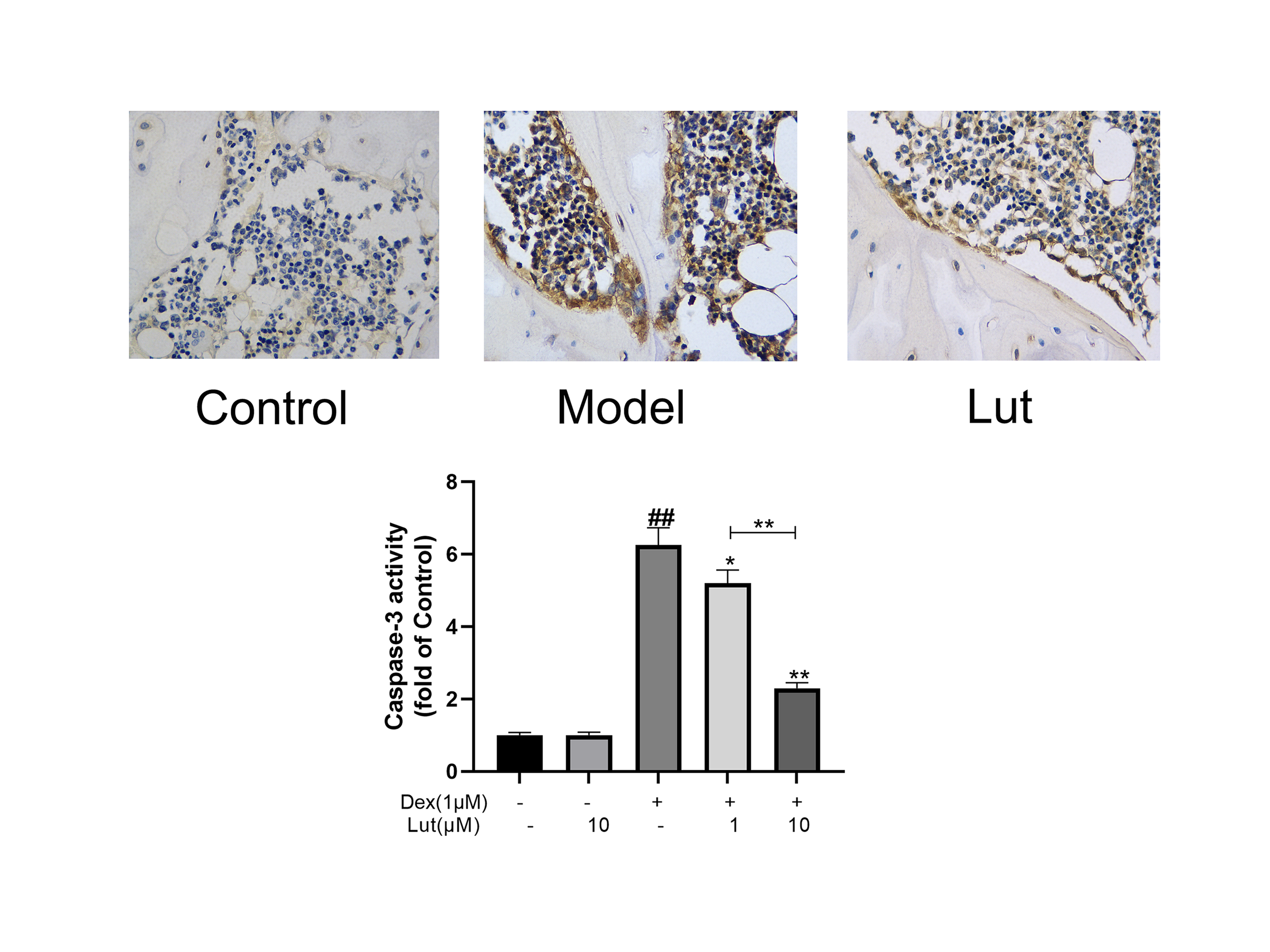

Supplement: Supplementary file 2 [file Image_2.tif]
